# Genetics tools for *corpora allata* specific gene expression in *Aedes aegypti* mosquitoes

**DOI:** 10.1038/s41598-022-25009-4

**Published:** 2022-11-28

**Authors:** Marcela Nouzova, Marten J. Edwards, Matthew DeGennaro, Dennys Leyva, Lilian V. Tose, Francisco Fernandez-Lima, Fernando G. Noriega

**Affiliations:** 1 Department of Biological Sciences and Biomolecular Science Institute, Florida International University, Miami, FL 33199 USA; 2 Institute of Parasitology, Biology Center of the Academy of Sciences of the Czech Republic, 37005 České Budějovice, Czech Republic; 3 Department of Biology, Muhlenberg College, Allentown, PA 18104 USA; 4 Department of Chemistry and Biochemistry and Biomolecular Science Institute, Florida International University, Miami, FL 33199 USA; 5 Department of Parasitology, University of South Bohemia, České Budějovice, Czech Republic

**Keywords:** Genetic engineering, Entomology

## Abstract

Juvenile hormone (JH) is synthesized by the *corpora allata* (CA) and controls development and reproduction in insects. Therefore, achieving tissue-specific expression of transgenes in the CA would be beneficial for mosquito research and control. Different CA promoters have been used to drive transgene expression in *Drosophila*, but mosquito CA-specific promoters have not been identified. Using the CRISPR/Cas9 system, we integrated transgenes encoding the reporter green fluorescent protein (GFP) close to the transcription start site of juvenile hormone acid methyl transferase (JHAMT), a locus encoding a JH biosynthetic enzyme, specifically and highly expressed in the CA of *Aedes aegypti* mosquitoes. Transgenic individuals showed specific GFP expression in the CA but failed to reproduce the full pattern of *jhamt* spatiotemporal expression. In addition, we created GeneSwitch driver and responder mosquito lines expressing an inducible fluorescent marker, enabling the temporal regulation of the transgene via the presence or absence of an inducer drug. The use of the GeneSwitch system has not previously been reported in mosquitoes and provides a new inducible binary system that can control transgene expression in *Aedes aegypti*.

## Introduction

The *corpora allata* (CA) is the site of synthesis of juvenile hormone (JH), an essential sesquiterpenoid that controls development and reproduction in insects^1^. In adult female mosquitoes, the biosynthetic activity of the CA is regulated by the interaction of multiple factors that stimulate (allatotropins) or inhibit (allatostatins) CA activity^1–3^. Developing a system to drive inducible CA-specific expression in mosquitoes would facilitate the study of modulatory factors of JH synthesis, as well as contribute to the identification of targets for designing new, specific, and affordable strategies suitable for mosquito control^4,5^. For instance, mosquito lines that ectopically express endogenous/foreign genes or dsRNAs could be generated, resulting in developmental or reproductive changes. Dissecting the roles of these critical genes requires manipulation of their functions in different tissues and at different developmental stages, which can be technically challenging.

An extensive collection of genetic tools supports the activity of researchers working with *Drosophila*^6,7^. Among them, the GAL4 enhancer trap system was used to identify the *Aug21* locus, described later as a CA-specific driver in the larval stages of *Drosophila*^8^. Many of these genetic resources are now available for non-model organisms such as mosquitoes^9^. Spatial control of gene expression in mosquitoes has been implemented in tissues such as olfactory neurons, midgut, and fat-body^10–13^. However, approaches to achieving mosquito CA-specific expression have not yet been described.

Binary systems have been previously used in mosquitoes, such as the GAL4/UAS and QF2/QUAS^11,13^. Still, more are needed for intersectional strategies that require the expression of multiple transgenes in distinct or overlapping groups of cell types. In addition to cell-specificity, achieving temporal control of transgenes adds another level of complexity over constitutive expression. Tetracycline-controlled Tet-Off and Tet-On gene expression systems have been used in several insect systems, including *Drosophila* and mosquitoes^14,15^. Due to the potential "off-target" effects of tetracycline and its analogs^16^, and a certain level of promoter "leakiness," the inducible GeneSwitch system could offer improvements. This technique is a modified version of the classic GAL4/UAS system, which allows inducible gene expression in *Drosophila* under the control of the progesterone analog RU-486^17,18^.

In the present study, we tested the hypothesis that we could link the activity of a strong CA promoter to drive the CA-specific expression of GFP in *Aedes aegypti* mosquitoes. We targeted the transcription start site of juvenile hormone acid methyl transferase (JHAMT), a JH biosynthetic enzyme that is specifically and highly expressed in the adult female CA^19,20^. Using the CRISPR/Cas9 system, we integrated a promoter-less transgene encoding eGFP (enhanced GFP) close to the transcription start site of the *jhamt* locus. Transgenic mosquitoes showed specific eGFP expression in the CA. In addition, CRISPR/Cas9 was also used to develop GeneSwitch driver and responder mosquito lines expressing an inducible fluorescent marker, enabling the temporal regulation of GFP expression via the presence or absence of RU-486.

Our lines offer a model to investigate the spatial and temporal patterns of the *jhamt* promoter. In addition to basic mosquito physiology research, the lines provide valuable genetic tools to investigate CRISPR-based technologies for mosquito population control. Overall, the findings and resources reported here expand the genetic toolkit for mosquito research.

## Results

### Construction of a plasmid vector for CRISPR/Cas9-mediated mutagenesis

We first tested the hypothesis that we could link the activity of a robust CA-specific promoter to drive the CA-specific expression of a transgene. We employed CRISPR/Cas9-mediated homology-dependent repair (HDR) to introduce an eGFP marker into the first exon of the *Ae. aegypti jhamt* locus as previously described^21^. Subsequently, the expression of eGFP was monitored to evaluate if the transgene would display the spatiotemporal features of expression of the *jhamt* gene.

**Figure 1:**
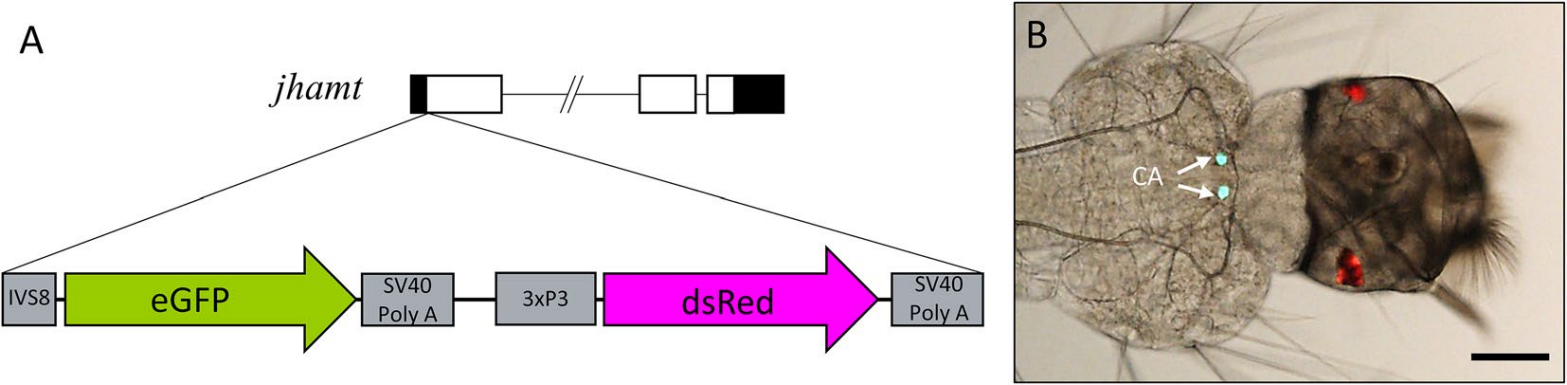
Integration of a promoter-less transgene encoding *eGFP* into the *jhamt* locus. (**A**) Diagram of the pSL_jhamt_eGFP construct. eGFP was flanked by the synthetic intron IVS8. dsRed (RFP) was used as a selection marker under the control of 3xP3, which drives expression in the eye. The insert was surrounded by two 1.5 kb *jhamt* locus homologous arms to facilitate recombination. (**B**) Image of head and thorax of a first instar larvae showing expression of dsRed in eyes and eGFP in the CA. Scale bar, 100 µm.

The plasmid pSL1180 (Amersham, UK) was used as a backbone to make the pSL_*jhamt*_*eGFP* construct. Integration of the transgene was guided into the first exon of the *jhamt* locus with two homologous sequences flanking the CRISPR target sites, a 1573 bp *jhamt* left arm and a 1511 bp *jhamt* right arm. The inserted cassette comprised a 1008-bp *eGFPSV40* promoter-less green fluorescent reporter and a 1241-bp fragment containing 3xP3dsRedSV40, a fluorescent red eye-specific marker used for visualization and selection of transformants^11^ (Fig. 1A). A synthetic intron IVS8 sequence was included in the first exon of *jhamt* prior to the start codon to enhance mRNA nuclear export.

To generate *jhamt* mutants, embryos of *Ae. aegypti* (Orlando strain) were injected with the pSL_*jhamt*_*eGFP* construct^21^. *jhamt*^*−/−*^ mutants never survived to adulthood but died as L4 instar just before pupation^21^. A stable heterozygous *jhamt*^+*/−*^ line was established after six backcrosses to wild type (WT) mosquitoes^21^. Transformants expressed dsRed in the eyes and eGFP in the CA (Fig. 1B).

### Tissue-specific expression of eGFP recapitulates jhamt expression

**Figure 2:**
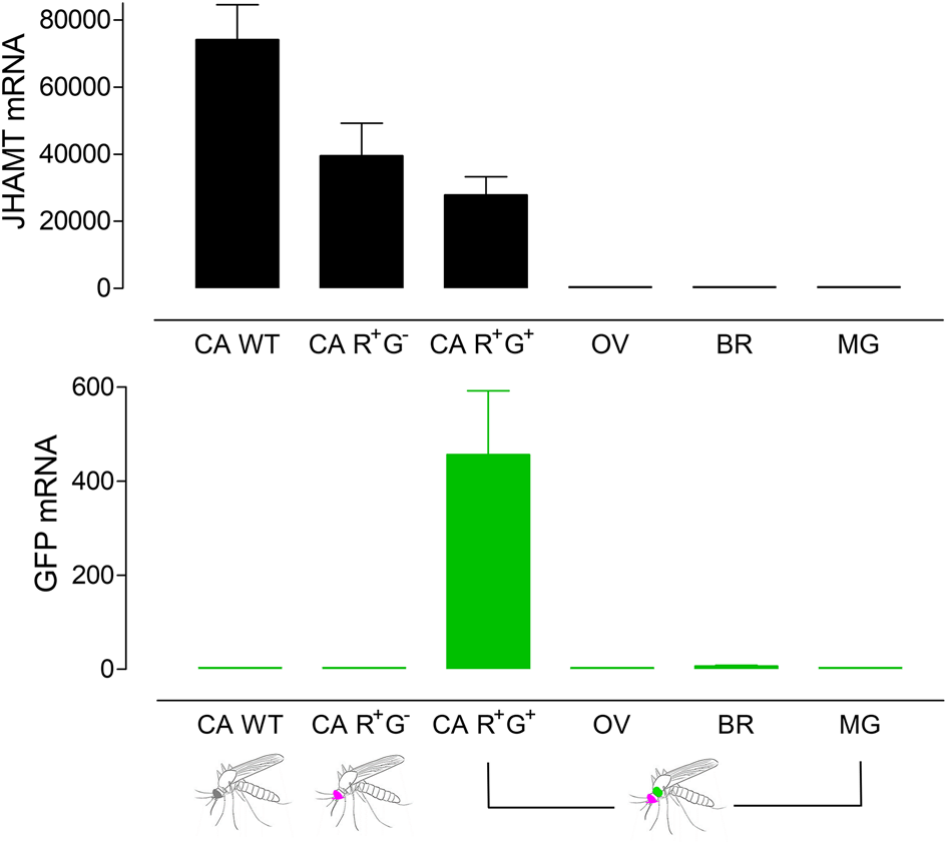
Tissue-specific expression of *jhamt* and *eGFP*. Real-time qPCR measurement of *jhamt* and *eGFP* mRNA levels in tissues of 3–5 days old sugar-fed WT and *jhamt*^*eGFP*+*/−*^ adult female exhibiting either eGFP^+^ or eGFP^−^ CA. CA WT: *corpora allata* wild-type; CA R^+^G^−^: heterozygote with red eyes and CA not green; CA R^+^G^+^: heterozygote with red eyes and green CA; *OV* ovaries, *BR* brain, *MG* midgut (all tissues dissected from insects with green CA). Levels of mRNAs are expressed as copy numbers per 10,000 copies of RpL32 mRNA. Each data point is an average of 3 independent biological replicates of tissues from 5 to 10 insects.

Real-time quantitative PCR (RT-qPCR) was used to analyze if *eGFP* expression recapitulates the transcript tissue specificity expression of *jhamt*^19,20^ (Fig. 2). *eGFP* and *jhamt* mRNA titers were evaluated in different tissues of WT and heterozygous *jhamt* adult female mutants exhibiting either eGFP^+^ or eGFP^-^ *corpora allata*. As previously described, *jhamt* mRNA expression was very high in the CA, with trace amounts present in the brain, ovaries, and midgut^19,20^; likewise, substantial amounts of *eGFP* mRNA were only detected in CA, although transcripts levels were less than 1% of those detected for *jhamt* (Fig. 2).

**Figure 3:**
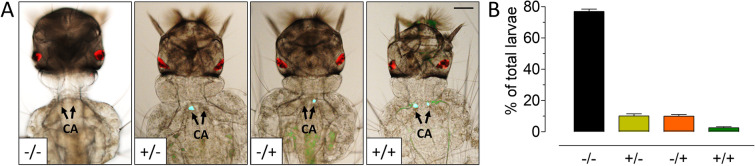
Asymmetric eGFP expression in CA: (**A**) Images of the four phenotypes of dsRed^+^ first instar larvae expressing eGFP in the CA. −/−: both CA GFP negative; +/−: only right CA eGFP positive; −/+: only left CA eGFP positive; +/+: both CA eGFP positive. Scale bar, 100 µm. (**B**) Percentages of individuals expressing eGFP in the CA. −/−: both CA eGFP negative, +/−: only right CA eGFP positive, −/+: only left CA eGFP positive. +/+: both CA eGFP positive. Scale bar, 100 µm.

To further evaluate the spatial expression of eGFP, the offspring of twenty-five eGFP^+^ females of G_7_–G_10_ generations (n = 1922, first instar larvae) were analyzed. Surprisingly, we detected four different "bilaterally asymmetric" phenotypes: insects (1) with both CA eGFP^−^ (77%), (2) with only a left CA eGFP^+^ (10%), (3) with only a right CA eGFP^+^ (10%) and (4) with both CA eGFP^+^ (3%) (Fig. 3).

**Figure 4:**
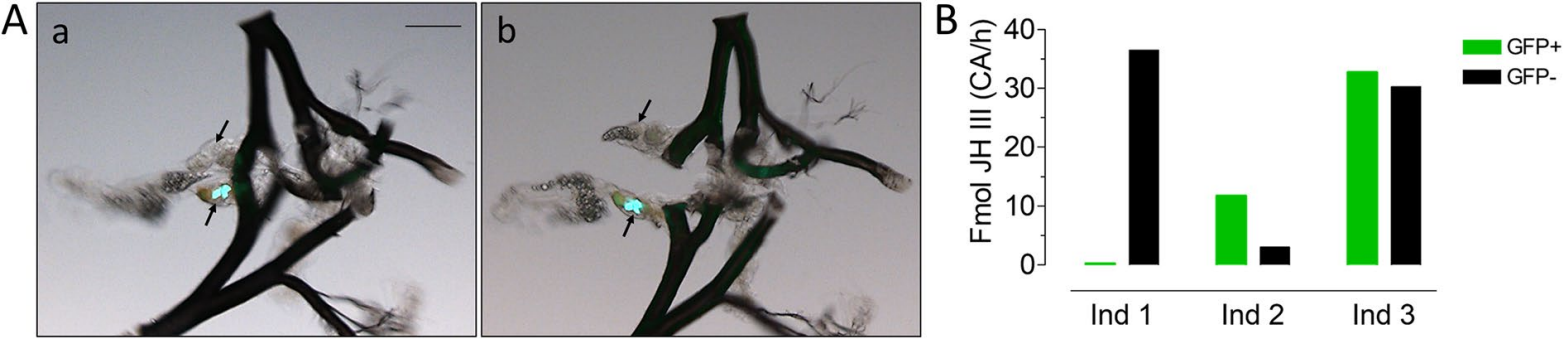
Lack of correlation between expression of eGFP and JH biosynthetic activity. Individual CAs were dissected from three insects expressing eGFP in only one CA, and JH biosynthesis was evaluated individually in each individual member of a CA pair. (**A**) Images of dissected CA, (a) before and (b) after separation of individual CA. (**B**) Amount of synthesized JH expressed as fmol of JH III/CA/h. Green bars: CA from GFP^+^, Black bars: CA from GFP^-^. Scale bar, 100 µm.

We subsequently evaluated the relationship between the expression of eGFP by each individual member of a CA pair, and each member's ability to synthesize JH in our ex vivo assay^22^. eGFP positive and eGFP negative CAs were dissected (Fig. 4a), and the amount of JH III synthesized by each individual gland ex vivo was analyzed by LC–MS/MS^23^. There was no correlation between the ability of the CA to synthesize JH and the eGFP expression in an individual CA (Fig. 4b).

### Temporal profile of eGFP expression

**Figure 5:**
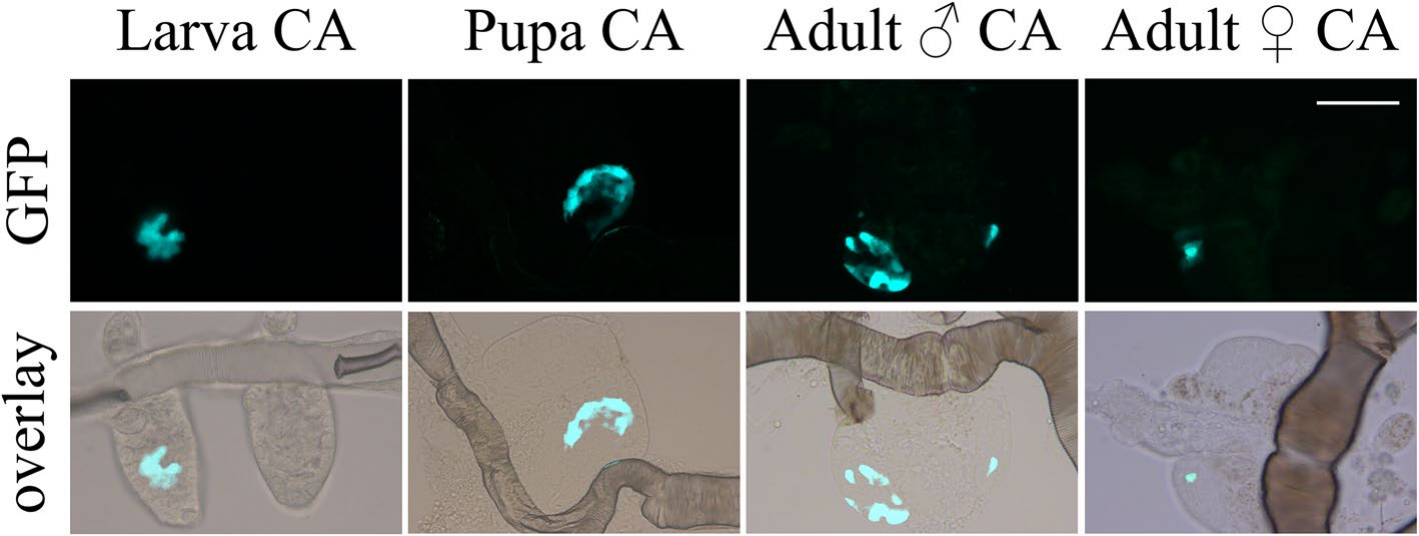
Stage-specific expression of eGFP. Fluorescent microscopy was employed to detect eGFP in the CA at all stages of the mosquito life cycle. Individual eGFP-positive CA were observed through the cuticle of larvae. Afterward, CA were dissected from late 4th instar larva, pupa, or adults to analyze in detail the presence of eGFP. eGFP was present only in some cells of the CA. Autofluorescence was detected neither in the CA of WT nor in most cells of the CA of mutants. Scale bar, 50 µm.

Levels of *jhamt* mRNA in WT are high in early 4th instar larvae, undetectable in late 4th instar and pupa, elevated in sugar-fed adult females, and low again in blood-fed females^19–21^. Using a fluorescent microscope, we evaluated the expression of eGFP during the life cycle of *jhamt*^+*/−*^ mosquitoes. We detected expression of eGFP in all the stages examined, including late 4th instar larvae and pupae, two periods where *jhamt* mRNA expression is never detected (Fig. 5). Theoretically, the fluorescent protein reporter should exhibit complete concordance with the expression of the endogenous gene, except perhaps in cases in which reporter visualization is due to eGFP perdurance^24^. To evaluate this possibility, we selected pupae expressing GFP and evaluated *eGFP* mRNA titers. We did not detect *eGFP* mRNA in early pupae that were GFP positive, confirming that the reporter visualization was due to GFP perdurance (Supp. Fig. 1).

To evaluate if the "asymmetric" expression of eGFP changed during development, we followed the expression of eGFP in 12 individual mosquitoes during their entire life cycle (n = 3 for each of the four phenotypes). We established that the phenotype of eGFP expression remained constant within but not across mosquitoes; a particular insect expressed eGFP in one (left or right), both or none of the CA from larvae to adult. In addition, the expression of eGFP was sporadic within the CA. Fluorescence was detected only in some cells of the CA, with the number of eGFP-positive cells varying significantly among individual transgenic animals (Fig. 5).

### Development of an inducible GeneSwitch system (GS)

**Figure 6:**
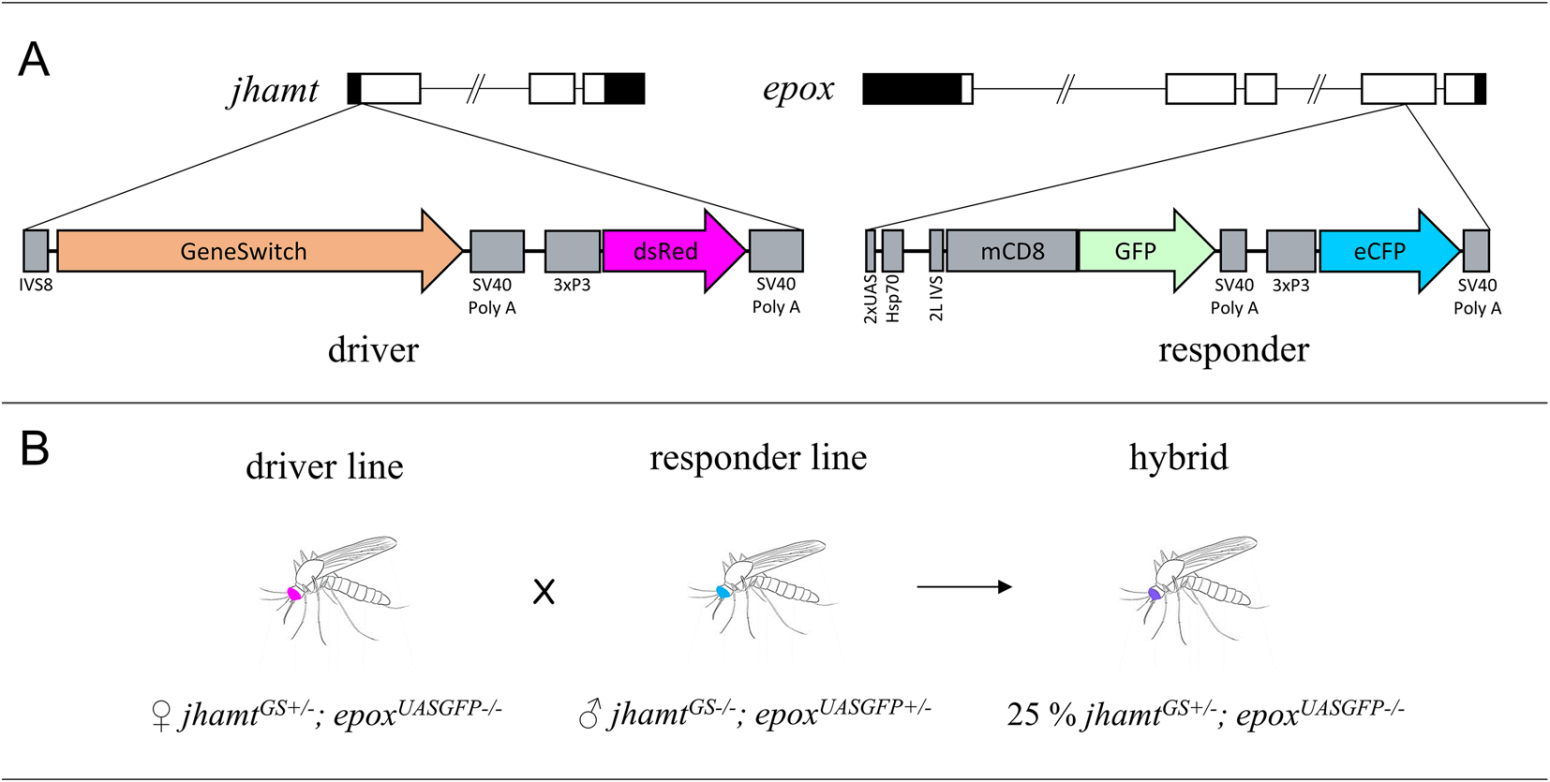
Development of an inducible GeneSwitch system. (**A**) Diagram of the driver and responder constructs. Driver: *GenSwitch* locus. dsRed (red eyes) was used as a selection marker under the control of 3xP3. The insert was surrounded by two 1.5 kb *jhamt* locus homologous arms to facilitate recombination. Responder: 2x*UAS* locus. GFP with a membrane localization signal (mCD8). eCFP (blue eyes) was used as a selection marker under the control of 3xP3. The insert was surrounded by two 1.5 kb *epox* locus homologous arms to facilitate recombination. (**B**) Crossing schemes to generate a hybrid. *jhamt*^*GS*+*/−*^ females were crossed with *epox*^*UASGFP*+*/−*^ males to generate hybrids containing both the effector and responder genes (*jhamt*^*GS*+*/−*^; *epox*^*UASGFP*+*/−*^). Hybrids were used to test inducibility by RU486.

CRISPR/Cas9 was also used to develop driver and responder mosquito lines expressing a transgene that could be activated by providing an inducer drug, RU486. The driver line was transformed with a pSL_*jhamt*_*GS* plasmid, which was also integrated into the first exon of the *jhamt* locus using the identical *jhamt* homologous sequences employed for the pSL_*jhamt*_*eGFP* construct, but with the *eGFP* sequence replaced by a *GeneSwitch Gal4* driver sequence (Fig. 6A). Transformants expressed dsRed in the eyes using the 3xP3 promoter.

To build the responder line, we integrated a transgene into the fourth exon of the *epox* locus, which encodes the methyl farnesoate epoxidase (EPOX). EPOX is another JH biosynthetic enzyme that is specifically and highly expressed in the CA^20,25^. The pSL_2xUAS_mCD8GFP_3xP3eCFP plasmid was modified to include *epox* homologous sequences flanking the CRISPR target sites, a 1540 bp (left) *epox* arm and a 1508 bp (right) *epox* arm^21^ (Fig. 6A). A stable heterozygous *epox*^*UASGFP*+*/−*^ line was established after six backcrosses to wild-type (WT) mosquitoes. Transformants expressed CFP in the eyes.

**Figure 7:**
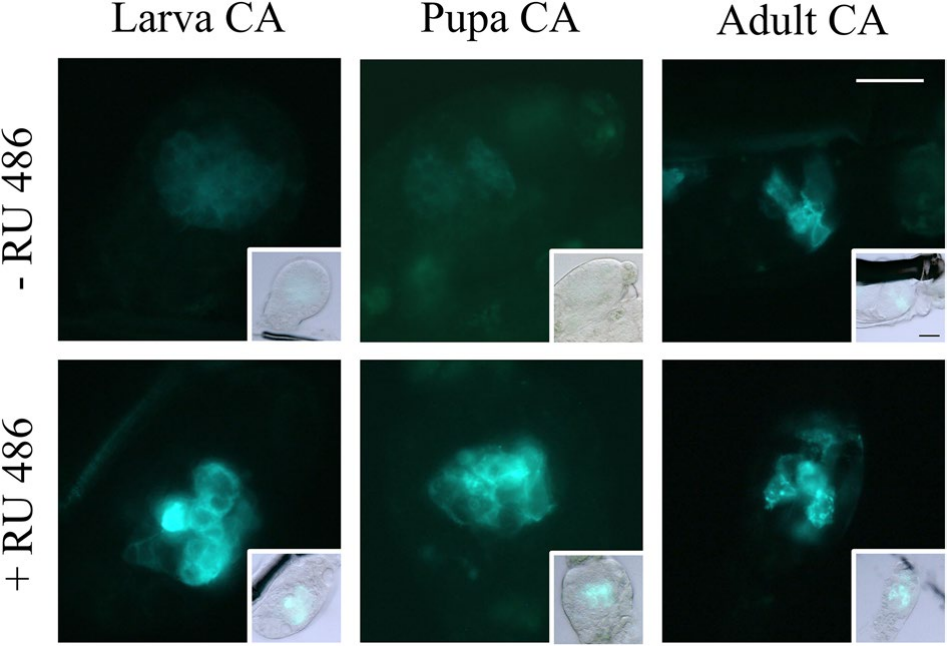
Induction of GFP expression. Fluorescent images show GFP expression in CA from 4th instar larva, pupa, and female adults without (−) or with (+) RU486 induction. Inset shows bright field images overlaid with fluorescence. Scale bar, 25 µm.

A homozygous driver line could not be established since *jhamt*^*GS/GS*^ insects died as 4th instar larvae. In addition, the homozygous responder line *epox*^*UASGFP/UASGFP*^ had severe physiological defects^21^; therefore, heterozygous driver and responder lines were used to evaluate the inducibility of UAS-driven GFP by RU486. Since the expression of eGFP in the CA of *jhamt *^*eGFP*+*/−*^ insects was sporadic, and only about 25% of the insects displayed eGFP^+^, RT-qPCR could not be used to assess the inducibility of *UASGFP* by RU486. Instead, the ability to induce GFP in 4th instar larvae, pupae, and adult mosquitoes was qualitatively evaluated using a fluorescent microscope. *jhamt*^*GS*+*/−*^ females were crossed with *epox*^*UASGFP*+*/−*^ males and F_1_ 1st instar larvae were screened for dsRed, and eCFP fluorescence in their eyes (Fig. 6B). Individual dsRed^+^ and eCFP^+^ insects were exposed to RU486 as either 4th instar larvae, pupae, or adults. We detected a strong GFP inducibility in 4th instar larvae and pupae with little background before induction. On the other hand, in adults, we also observed a weak GFP signal in non-induced animals (Fig. 7), indicating that the *GS* inducible expression system in these adult mosquitoes can be leaky. In all cases, we only observed GFP expression in a few adult female CA cells, a sporadic pattern similar to that observed in the *jhamt*^*eGFP*+*/−*^ line, confirming that the expression of *GS* inserted in the *jhamt* transcription start locus was regulated similarly to the expression of *eGFP* in the *jhamt *^*eGFP*+*/−*^ line.

## Discussion

A few tissue-specific promoters have been described in mosquitoes to drive the expression of transgenes in tissues such as the midgut^10^, salivary glands^26^, fat body^27^, and testis^28^; nevertheless, promoters that drive mosquito CA-specific expression have not been reported. In contrast, different promoters have been used to drive CA-specific expression in *Drosophila*, with the *jhamt* promoter displaying more robust CA-specific expression than the *Aug21* locus^29,30^. The present series of experiments used the CRISPR/Cas9 approach to assess the ability of the *jhamt* promoter to drive the specific expression of eGFP in the mosquito CA gland. We observed that the introduced transgene exhibited some of the features of endogenous gene expression. The tissue-specificity represented wild-type patterns of *jhamt* expression. Initially, we thought the temporal-specificity was lacking, as we observed the presence of GFP in pupae. Subsequently, we uncovered that the presence of GFP in the pupa stage resulted from eGFP perdurance^22^, because no de novo transcription of *jhamt* or *GFP* occurred at this stage. Perdurance is a common problem with in vivo reporters, and in some cases, the GFP signal lasted for about 4–5 days after the disappearance of the mRNA signal^31^. In summary, GFP perdurance could have confounded our assessment of the transgene expression in some stages.

In addition, the *eGFP* mRNA expression was much lower than *jhamt* expression, although the transgene expression was not particularly low. The strength of the native *jhamt* promoter is extremely high^20^. The amount of *jhamt* mRNA in the CA is seven times larger than the amount of ribosomal protein L32 mRNA (the expression level of *jhamt* is around 70,000 copies/10,000 copies of rpL32). The eGFP transgene was expressed at 500 copies/10,000 copies of rpL32. To provide some context, half of the JH synthetic enzyme pathway transcripts were expressed at the same or lower level than the eGFP transgene in the CA^20^. Nevertheless, the intensity of the epifluorescent signal allowed the visualization of the "green CA" through the cuticle of live larvae from first to fourth-instar and dissected CA from pupae and adult mosquitoes.

In the locust *Schistocerca gregaria*, JH biosynthesis by the paired CA gland was described as remarkably "asymmetric" with respect to the relative JH synthetic activity of either member of the pair^32^. This apparently random pattern was observed at all ages studied^32^. We also observed a bilateral asymmetry of eGFP expression in the two individual CA of a single insect, as well as an unequal distribution among the four "asymmetric phenotypes." The vast majority of larvae (~ 80%) did not show any fluorescent signal in either of the two glands, and only about 3% of the larvae were fluorescent in both glands. Remarkably, a particular phenotype remained invariable during the entire life cycle of an individual mosquito. We cannot explain the molecular basis or significance of this asymmetry. There was no correlation between the expression of *jhamt* and *GFP* in individual glands, which suggests a complex mechanism for *jhamt* expression, perhaps involving the spatial dynamics of *cis*-acting elements within the *jhamt* gene itself.

Expression of GFP was characteristically observed in a random cellular distribution within the CA that normally express *jhamt*. The local chromatin environment at the site of transgene insertion can alter both the pattern and the level of transgene expression^33,34^. Although integration sites in the genome might result in uniform expression in a particular cell type; often transgene expression is stochastic; that is, not all cells of a given type express the transgene, and the precise cells showing expression vary from individual to individual, frequently because of the difficulty in overcoming chromatin blocks to initiating transcription^33,34^. Mechanisms involving chromatin modification may be responsible for the silencing of genes in position effects resulting from the insertion of transgenes. The results presented here suggest the action of silencer-like elements affecting GFP expression at the chromosomal insertion site in a cell-specific manner in transformed mosquitoes.

It is also possible that the low and unpredictable levels of eGFP expression we observed were due to technical features of the construct and that the potential strength of the *jhamt* promoter was underestimated as a result. For example, a synthetic IVS8 sequence was included in the first exon of *jhamt* prior to the start codon because it is a standard component of the pSwitch system (Invitrogen). Short introns are routinely added to transgenes to enhance mRNA nuclear export in many eukaryotes, including *Drosophila* and *Aedes albopictus* C6/36 cells^34–36^. The IVS8 fragment included canonical eukaryotic donor and acceptor splice sites and their respective consensus flanking sequences^37^. The sequence of this fragment was verified in G4 mosquito lines, and no mutations were detected. However, proteins such as transcription factors or splicing factors that normally interact with the *jhamt* 5′-UTR could have conceivably interfered with the expected splicing of the IVS8 intron in such a way as to excise eGFP from the mature transcript, using an aberrant and perhaps unconventional 3′ splice site^38^. We tested if inappropriate splicing due to the presence of the artificial intron was reducing the observed transgene expression. Quite the opposite, the amount of transcribed 5'UTR upstream of IVS8 (Supl. Fig. 2) corresponded well to the amount of transcribed *eGFP* or transcribed *jhamt* (Fig. 2), and it was in agreement with the visual presence or absence of eGFP, detected using the fluorescence microscope. We concluded that the transgene expression was defined primarily by the transcription efficiency and not by incorrect splicing due to the presence of the IVS8 intron.

Many experiments in biological research rely critically on the ability to express exogenous proteins or RNAs in transgenic animals in a manner that is regulated for level, timing, and cell type. Enhancer trapping has been widely used to identify tissue-specific promoters in *Drosophila*^39^, often resulting in the reporter having spatial and temporal expression identical to the patterns of the associated enhancer region. A PiggyBac transposon-based enhancer-trap system was developed for the mosquito *Anopheles stephensi*^40^ and used to generate enhancer-trap lines expressing eGFP in adult female salivary glands, midgut, and fat body^40^. While *Drosophila* and *Anopheles* reporter genes have frequently been shown to respond to neighboring transcriptional regulatory elements, gene regulatory mechanisms may be more complex in *Ae. Aegypti*—with a genome size five times larger than either model system^41,42^. The longer intronic and intergenic sequences in *Aedes* may interfere with the ability of the transcriptional apparatus to co-opt the precise spatial and temporal dynamics of gene expression that are required for successful enhancer traps.

By inserting our eGFP construct within the open reading frame of the *jhamt* gene, we disrupted the structure of the locus. Although some fundamental elements of the *jhamt* promoter drove the transgene expression in the correct tissue (CA) at the expected developmental stages (adult females), other critical regulatory components were not preserved in our design.

Unexpectedly, the expression of eGFP in the CA of an individual female mosquito did not predict the expression of the reporter in her progeny. Having an eGFP^+^ or eGFP^-^ female parent did not change the proportion of eGFP^+^ in the offspring, suggesting that the pattern of GFP expression is defined de novo in each generation and may be influenced by epigenetic factors. The role of epigenetic factors in the regulation of the mosquito CA is currently unknown. Our mosquito lines could provide a model system to investigate epigenetic modifications that could underlie intergenerational differences in eGFP expression in *Ae. aegypti* CA.

The ultimate aim of these experiments was to drive CA-specific expression in mosquitoes with defined temporal and spatial patterns. For that reason, it would be desirable to develop a conditional expression system that is activated only by the presence of an inducer. GeneSwitch has been demonstrated to function in *Drosophila*^17^, albeit the system can be leaky depending on the nature of the promoter and responder transgene^18^. In our case, the expression of GFP in the absence of the inducer RU-486 was low in larvae and pupae, and the inducer clearly activated the reporter gene. In adults, the expression of GFP in the absence of an inducer was more noticeable. Thus, in cases where tight regulation is required, it might be advisable to explore a different system that has been validated in dipterans, including mosquitoes, such as the Q-system^43–45^ and T2A^46,47^.

In summary, we report a set of experiments in which we empirically tested a strategy to direct exogenous gene expression in the CA of mosquitoes. Our data suggest that we have not developed an unequivocally useful general tool to direct the expression of transgenes. Whether this is due to the approach, the construct, or a peculiarity of this particular locus would require additional studies. Nevertheless, given the widespread need for these methods, we expect our results will be a useful guide to generate improved approaches in the future. If the immediate goal were to use the *jhamt* promoter to drive the expression of transgenes in wild populations, the lines we generated are a first step in this process and require additional optimization. However, we have generated stable mosquito lines that provide tools for investigating the spatial and temporal patterns of the *jhamt* promoter. Although the concept of harnessing the tissue-specific regulation by inserting a promoter-less transgene inside a locus of a highly expressed CA gene did not render the expected spatiotemporal expression, our intriguing expression patterns revealed that *jhamt* is regulated by a complex interaction of promoter and enhancers that cannot be "captured" by approaches that drive specific expression in other mosquito tissues^10,25–27^. Nevertheless, our findings extend the genetic toolkit in *Aedes aegypti* by demonstrating the efficacy of the GeneSwitch binary system to control gene expression spatially and temporally. This technique can enhance our understanding of this important disease vector and can be applied to develop genetic mosquito control methods.

## Methods

### Insects

*Ae. aegypti* of the Orlando strain were reared at 28 °C and 80% humidity as previously described^48^. Adult mosquitoes were offered a cotton pad soaked in a 10% sucrose solution. Four-day-old female mosquitoes were fed pig blood equilibrated to 37 °C, and ATP was added to the blood meal to a final concentration of 1 mM immediately before use, as previously described^48^.

### CRISPR/Cas9 reagents and generation of *jhamt*^*dsRed*^ and *epox*^*eCFP*^ mutant lines

Homology-directed repair (HDR) was utilized to introduce gene cassettes encoding fluorescent markers into the first exon of *jhamt* and the fourth exon of *epox* genes, as previously described^21^. Stable germline integrations were generated by injecting CRISPR/Cas9 reagents into the posterior end of preblastoderm *Ae. aegypti* embryos^21^. One line with a verified insertion site was selected for each transgene, backcrossed to the WT strain for five generations, and used for experiments starting at G_6_^21^. Detailed steps of construct design are included as Supplemental Materials.

### Induction of GeneSwitch with RU-486

RU-486 was diluted in ethanol (1 mg/ml) and directly added to the water, where early 4^th^ instar larvae were raised at a final concentration of 1% ethanol (10 µg/ml RU-486).

To induce GeneSwitch in the adult mosquitoes, RU-486 was mixed with the sugar meal to get a final concentration of 1% ethanol (5 µg/ml RU-486) and provided *ad-libitum* to insects from eclosion. Control insects received the same concentration of ethanol (1%).

### Quantitative PCR (RT-qPCR)

Total RNA was isolated using the Norgen Biotek Total RNA purification kit, treated with DNase I, and reverse-transcribed using the Verso cDNA Synthesis Kit (Thermo Fisher). The number of mRNA copies normalized to rpL32 mRNA was quantified in triplicate reactions in a 7300 Real-Time PCR System using the TaqMan Universal PCR MasterMix (Applied Biosystems)^21^. Gene accession numbers and primer and probe sequences are listed in Table S1.

### JH biosynthesis assay

Mosquitoes were cold-anesthetized, and the CA complexes were dissected and incubated at 32 °C for 4 h in 150 μl of M-199 tissue culture medium (Gibco) containing 2% Ficoll, 25 mM Hepes (pH 6.5), and 100 μM methionine^22^. JH III in the tissue culture medium was analyzed by LC–MS/MS, as previously described^23^.

### Fluorescent microscopy

The patterns of eGFP, dsRed and eCFP expression were determined by microscopic observations of larvae, pupae, and adults using a fluorescent dissecting microscope equipped with optical filters for Cy3, eGFP, and simultaneous visualization of both colors. The evaluation of eGFP expression in CA was performed on unfixed preparations using a DM 5500 B Leica fluorescence microscope with a Leica DFC 310 FX mounted camera and Leica LAS imaging software. Autofluorescence was detected neither in the CA of WT nor in most cells of the CA of mutants.

## Supplementary Information

Supplementary Information.

## Data Availability

All data generated or analyzed during this study are included in this published article (and its Supplementary Information files).
