# Supplementary material for: Genetics tools for corpora allata specific gene expression in Aedes aegypti mosquitoes
Source: Sci Rep. 2022 Nov 28;12:20426. doi: 10.1038/s41598-022-25009-4 (PMC9705396; doi:10.1038/s41598-022-25009-4)

Supplementary information.

Title: Genetics tools for *corpora allata* specific gene expression in *Aedes aegypti* mosquitoes.

Marcela Nouzova[1,2*], Marten J. Edwards[3], Matthew DeGennaro[1], Dennys Leyva[4], Lilian V. Tose[4], Francisco Fernandez-Lima[4] and Fernando G. Noriega[1,5*]

[1]Department of Biological Sciences and Biomolecular Science Institute, Florida International University, Miami, FL, 33199, USA.

[2]Biology Center of the Academy of Sciences of the Czech Republic, Institute of Parasitology, 37005, České Budějovice, Czech Republic.

[3] Department of Biology, Muhlenberg College, Allentown, PA 18104, USA.

[4] Department of Chemistry and Biochemistry and Biomolecular Science Institute, Florida International University, Miami, FL 33199, USA.

[5]Department of Parasitology, University of South Bohemia, České Budějovice, Czech Republic.

*Corresponding authors: marcela.nouzova@paru.cas.cz, noriegaf@fiu.edu

Correspondence to: noriegaf@fiu.edu

Supplementary Materials and Methods

Construction of Plasmid Vectors for CRISPR/Cas9-Mediated *jhamt* and *epox* Mutagenesis. The individual parts of the construct were introduced using the In-Fusion® system from Takara Bio (Clontech, USA). In-Fusion primers were designed using SnapGene version 4.0.8 (GraphPad, IL). All intermediate products, as well as the final plasmid, were verified by sequencing. The plasmid pSL1180 (Amersham, UK) was used as a backbone to generate the donor DNA template for integration into exon 1 of the *jhamt* locus. The individual parts of the construct were introduced using the In-Fusion® system from Takara Bio (Clontech, USA). The pSL1180 plasmid was digested with *Xba*I, and fused with a 1241-bp fragment containing the 3xP3dsRedSV40 sequence from the pMos-3xP3dsRed-attp (Addgene #52904), using primers pSL-dsRed F and pSL-dsRed-PmeI R (Table S1). A *Pme*I site was introduced to facilitate cloning the homologous sequences near the *jhamt* integration site. The resulting pSL1180_3xP3dsRedSV40 plasmid was digested with *Nde*I, and fused to two fragments: a 201-bp IVS8-eGFP (from pSwitch, Invitrogen, primers pSLNdeI_IVS8_F and eGFP_IVS8_R), and a 1008-bp promoter-less eGFP-SV40 (from pSL {attP1-3xP3-eGFP-SV40-attP2} Acc #KC897089, primers IVS8_eGFP_F and pSLNdeI_eGFP_R). This new plasmid was named pSL_IVS8eGFPSV40_3xP3dsRedSV40. To integrate into exon 1 of *jhamt*, the pSL_IVS8eGFPSV40_3xP3dsRedSV40 was modified to include homologous sequences flanking the CRISPR target sites. The 1573 bp upstream (left) arm and 1511 bp downstream (right) arm were amplified from the Orlando strain *Ae. aegypti* genomic DNA using JHAMT_Upstream_F/R and dsRed_JHAMT_DS_F/R primers (Table S1). The left homologous arm was inserted into the *Pac*I digested plasmid pSL_IVS8eGFPSV40_3xP3dsRedSV40. This modified plasmid was digested with *Pme*I, and the right homologous arm was incorporated. The final plasmid pSL_JHAMT_eGFP (donor DNA) was purified for embryo injections from *E. coli* using the Endo-free Maxiprep DNA isolation kit (QIAGEN, USA).

Similarly, the plasmid pSL1180 (Amersham, UK) was used as a backbone to generate the donor DNA template for integration into exon 4 of *epox*. pSL1180 was linearized with *Age*I, and the following four fragments were joined using In-Fusion. Fragment 1 (424 bp), containing 2xUAS and a *D. melanogaster* intervening sequence (DMIVS), was amplified from the pJFRC2-10XUAS-IVS-mCD8::GFP plasmid (Addgene #26214), using the primer 1F (which added a *Pac*I site to the 5' end of the amplicon), and the primer 1R (which added a *Sgr*DI site to the 3' end) (Table S1). Fragment 2 (522 bp), containing an SV40 polyadenylation signal, the 3xP3 enhancer, and a *D. melanogaster hsp70* basal promoter, was amplified from the above described pSL_IVS8GFPSV40_3xP3dsRedSV40 plasmid with primers 2F and 2R. Fragment 3 (735 bp), containing the eCFP gene (minus the nuclear localization signal), was amplified from the pSL1180-HR-PUb-eCFP plasmid (Addgene #47917) with primers 3F and 3R. Fragment 4 (166 bp), containing the SV40 polyadenylation signal, was amplified from the pSL1180-HR-PUb-eCFP plasmid (Addgene #47917), with primers 4R and 4R, which added a *Pme*I restriction site to the 3' end of the amplicon (Table S1). The resulting pSL_2xUAS_3xP3_eCFP plasmid was

linearized with *Sgr*DI, and a fragment of 1428 bp, containing the mCD8-GFP sequence amplified from the plasmid PUb-mCD8-GFP-T2A-dsRed-NLS-SV40 (Addgene #130665) using primers 5F and 5R, was inserted using In-Fusion to produce pSL_2xUAS_mCD8GFP_3xP3eCFP. To integrate into exon 4 of *epox*, the pSL_2xUAS_mCD8GFP_3xP3eCFP plasmid was modified to include homologous sequences flanking the CRISPR target sites. The 1540 bp upstream (left) arm and the 1508 bp downstream (right) arm were amplified from *Ae. aegypti* Orlando strain genomic DNA, using the EPOX_Upstream_F/R and EPOX_Downstream_F/R primers, respectively (Table S1). The left homologous arm was incorporated into the pSL_2xUAS_mCD8GFP_3xP3eCFP plasmid digested with *Pac*I. The resulting plasmid was digested with *Pme*I, and the right homologous arm was incorporated. The final plasmid named pSL_EPOX_UAS_GFP (donor DNA) was purified for embryo injections from *E. coli* using the EndoFree Maxiprep DNA isolation kit (QIAGEN, USA).

Similarly, to generate the donor DNA pSL_JHAMT_GS template for integration into exon 1 of the *jhamt* locus, plasmid pSL1180 (Amersham, UK) was again used as a backbone. First pSL1180 was digested by Xba I, and fused with a 1241 bp fragment containing 3xP3dsRedSV40 from pMos-3xP3DsRed-attp (*Addgene* #52904) (primers pSL-DsRed F and pSL-DsRed-PmeI R were used)(Table S1). During this step, a unique side for Pme I was introduced to facilitate cloning homologous sequences surrounding the *jhamt* integration site. The resulting plasmid was named pSL1180_3xP3dsRedSV40. pSL1180_3xP3dsRedSV40 was digested with Nde I, and fused with two fragments, IVS8-Switch (2181 bp, from pSwitch, (Invitrogen, USA), primers pSLNdeI_IVS8_F and SV40_Switch_R), and SV40 (276 bp, from pMos-3xP3DsRed-attp *Addgene* #52904, primers Swith_SV40_F and pSLNdeI_eGFP_R) (Table S1). This plasmid was named pSL_IVS8SwitchSV40_3xP3dsRedSV40. To integrate it into exon 1 of the *jhamt* locus, pSL_IVS8SwitchSV40_3xP3dsRedSV40 was modified to include homologous sequences surrounding the CRISPR target sites. The 1573 bp upstream (left) arm and the 1511 bp downstream (right) arm were amplified from Orlando strain *Ae. aegypti* genomic DNA using these primers: JHAMT_Upstream_F/JHAMT_Upstream_R and DsRED_JHAMT_DS_F/ DsRED_JHAMT_DS_R (Table S1). The left homologous arm was incorporated into pSL_IVS8SwitchSV40_3xP3dsRedSV40 digested by the Pac I enzyme. The resulting plasmid was digested with Pme I, into which the right homologous arm was incorporated. The final plasmid was named pSL_jhamt_GS (donor DNA). The final plasmid DNA for embryo injection was purified from *E. coli* using the EndoFree Maxiprep DNA isolation kit (QIAGEN, USA).

Supplementary Figure 1: The fluorescence of eGFP in early pupae is caused by the perdurance of eGFP protein and not by *de novo* mRNA transcription.

Hypothesis: The visualization of the reporter protein in early pupae is due to eGFP perdurance.

Rationale of the experiment: We selected individuals expressing the fluorescent reporter protein in the CA using a fluorescent microscope. *jhamt* transcripts are absent in early pupae but present in late pupae and adults[1,2,3]. If the expression of the transgene, driven by the *jhamt* promoter, follows the temporal expression of the endogenous gene, we should not detect *eGFP* mRNA in the early pupae. Therefore, the visualization of the reporter protein in early pupae would be due to eGFP perdurance.

Results and conclusion: CA were dissected from early pupae (12-24 hours after pupation), late pupa (36-48 hours after pupation), and four days old sugar-fed adults (4d SF). Transcript levels of *eGFP*, *jhamt*, and *rpL32* (ribosomal protein L32, a housekeeping gene used as a control) were analyzed by RT-PCR. *eGFP* transcripts were present in late pupae and adult CA. The *eGFP* transcripts were absent in early pupae, even if the eGFP protein was present. In summary, *eGFP* transcript levels correlated with the expression of *jhamt* mRNA. The variability of *eGFP* transcript amounts among individual samples from the same stage is due to a variable number of cells expressing *eGFP* in each CA (see Fig. 5). Our data support the hypothesis that the presence of eGFP in early pupae is caused by perdurance of the protein and not by *de novo* mRNA transcription.

Supp. Fig. legends: Agarose gel analysis of the PCR amplified *eGFP* and *jhamt* transcripts. mRNA was extracted from CA of three stages of $jhamt^{+/-}$ heterozygous mutants (n=5; three biological replicates). Early pupa: CA were dissected from pupae 12-24 hours after pupation. Late pupa: CA were dissected from pupae 36 - 48 hours after pupation. 4d SF adult: CA were dissected from four-day-old sugar-fed adults. The presence of eGFP protein in the CA was visually verified by fluorescence microscopy. Transcript levels were analyzed by RT-PCR. Amplification of the ribosomal protein L32 gene was used as an internal standard. **Upper gel:** Levels of *eGFP* transcripts in the three types of CA samples. *eGFP* transcripts are present in late pupae and adult CA. The *eGFP* transcripts are absent in early pupa, even if eGFP protein was present. **Middle gel:** Levels of *jhamt* transcripts in the three types of CA samples. *jhamt* transcript is present in late pupae and adult CA. **Lower gel** shows consistent levels of *rpL32* expression across all analyzed samples.

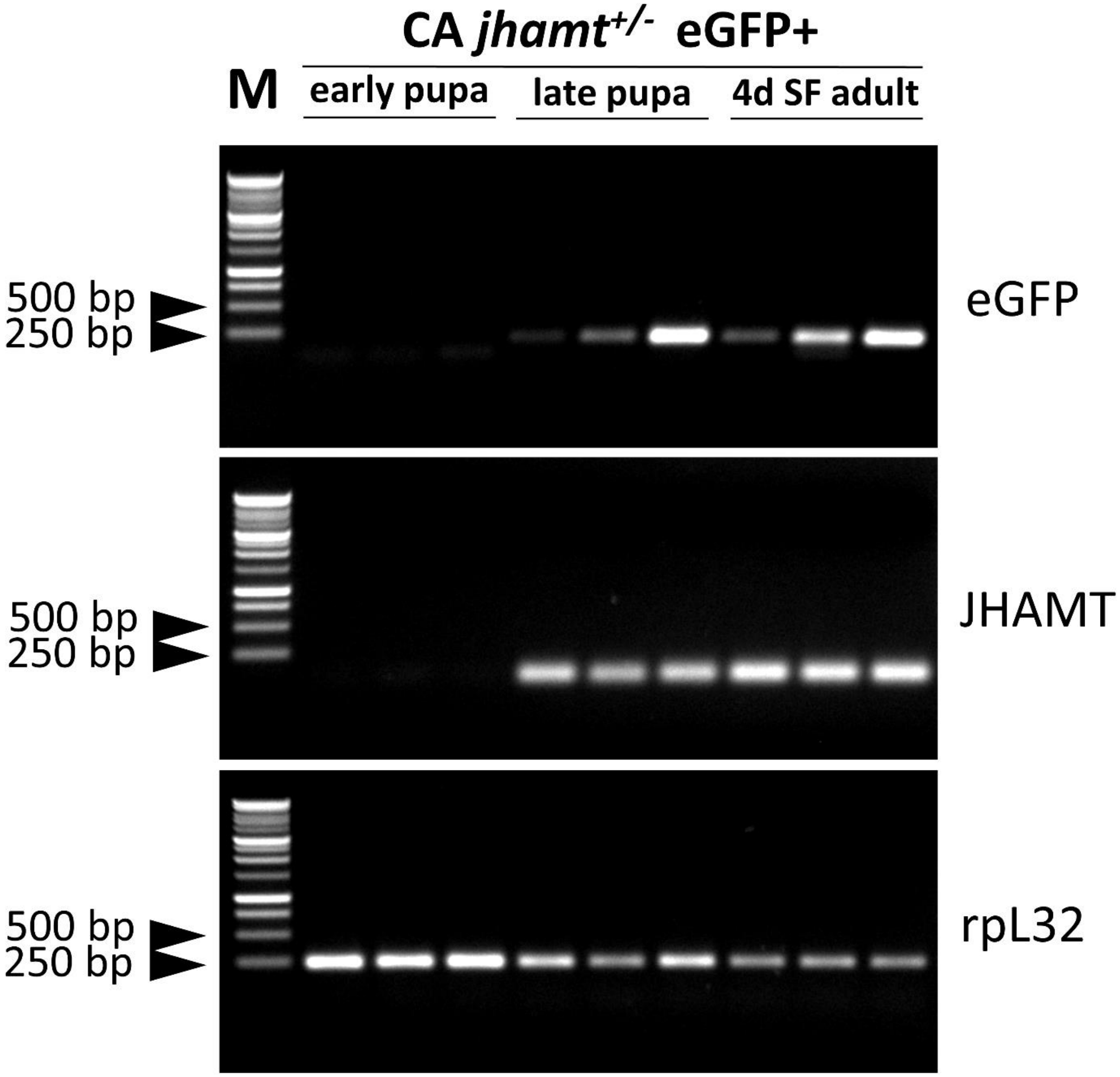
CA jhamt+/- eGFP+
M
early pupa
late pupa
4d SF adult
500 bp
250 bp
eGFP
500 bp
250 bp
JHAMT
500 bp
250 bp
rpL32

Supplementary Figure 2: Evaluation of alternative splicing due to the presence of the artificial intron IVS8 between the *jhamt* transcription start and the *eGFP* transgene.

Hypothesis: Inappropriate splicing due to the presence of the artificial intron reduces the transgene expression.

Rationale of the experiment: We used different primer combinations to test transcript levels of the *jhamt* locus in CA of three types of 2nd instar larvae: wild type (*wt*), *jhamt*$^{-/-}$ mutants with visible eGFP fluorescence (eGFP+), and *jhamt*$^{-/-}$ mutants without visible eGFP fluorescence (eGFP-). Two primer combinations were employed. 1) Primers 5UTR F and R should generate a PCR product size of 70 bp that represent the transcript levels of the *jhamt* locus in the different CA types. 2) Primers 5UTR F and eGFP R should generate a PCR product size of 203 bp in *jhamt*$^{-/-}$ mutants if the intron is normally spliced, or alternatively, a PCR product size of 321 bp in *jhamt*$^{-/-}$ mutants if the intron is not spliced. Wild-type larvae that do not carry the transgene should not generate any PCR products.

Results and conclusion: The amounts of transcribed 5'UTR upstream of IVS8 corresponded well to the amounts of transcribed *eGFP* or transcribed *jhamt* (Fig. 2). Transcript levels were in agreement with the visual presence or absence of eGFP, detected using the fluorescence microscope. In *jhamt*$^{-/-}$ mutants, the combination of primers 5UTR F and eGFP R always generated a PCR product size of 203 bp. Based on the results obtained using both primer combinations, we concluded that the transgene expression was defined primarily by the transcription efficiency and not by incorrect splicing due to the presence of the IVS8 intron.

A: Diagram of the 5' transgene insertion site in the *jhamt* locus, with the position of the primers used for transcript analysis. The diagram displays the 5'UTR of the endogenous *jhamt* gene (blue), 21bp of the endogenous *jhamt* coding sequence (orange), the inserted intron IVS8 (grey), and a part of the inserted *eGFP* coding sequence (green). The starting positions of each segment relative to the beginning of the *jhamt* 5'UTR are labeled by numbers below the diagram. Blue arrows depict primers with their 5' starting position (numbered).

B: Diagrams of the potential PCR product sizes obtained using the different primer combinations. Sizes of the PCR products (number above). Primer combinations are shown below the diagram of each product.

C: Agarose gel analysis of the PCR amplified 5'UTR region using different primer combinations. mRNA was extracted from three categories of 2nd instar larvae (n=5): *wt*, *jhamt*$^{-/-}$ mutants with visible eGFP fluorescence (eGFP+), and *jhamt*$^{-/-}$ mutants without visible eGFP fluorescence (eGFP-). The presence or absence of eGFP in the CA of each larva was visually verified by fluorescence microscopy. Transcript levels were analyzed by RT-PCR. Amplification of the ribosomal protein L32 gene was used as an internal standard. **Upper gel:** Levels of *jhamt* 5'UTR transcripts in the three categories of larval samples. Levels of *jhamt* 5'UTR expression are high in *wt* larvae. In these larvae, *jhamt* expression is typically high. In *jhamt*$^{-/-}$ mutants, where eGFP is not visually present (eGFP-), the *jhamt* 5'UTR PCR product is not detectable. In *jhamt*$^{-/-}$

mutant larvae in which eGFP is visible in CA (eGFP+), expression is detectable but at much lower levels than the *jhamt* 5'UTR expression in *wt*. These PCR product sizes were the expected 70 bp (white arrowhead). **Middle gel:** Levels of *jhamt* 5'UTR plus *eGFP* transcripts. No PCR product was detected in *wt* larvae (without the eGFP insertion). In *jhamt*$^{-/-}$ mutants, the levels of 5'UTR plus *eGFP* transcripts corresponded to the levels of *jhamt* 5'UTR transcripts observed in the upper gel (i.e., not detectable in eGFP- mutants and low in eGFP+ mutants). The 5'UTR F and eGFP R primer PCR product sizes were 203 bp, indicating that the IVS8 intron were appropriately spliced out in eGFP+ *jhamt*$^{-/-}$ mutants. **Lower gel** shows consistent levels of *rpL32* expression across all analyzed samples.

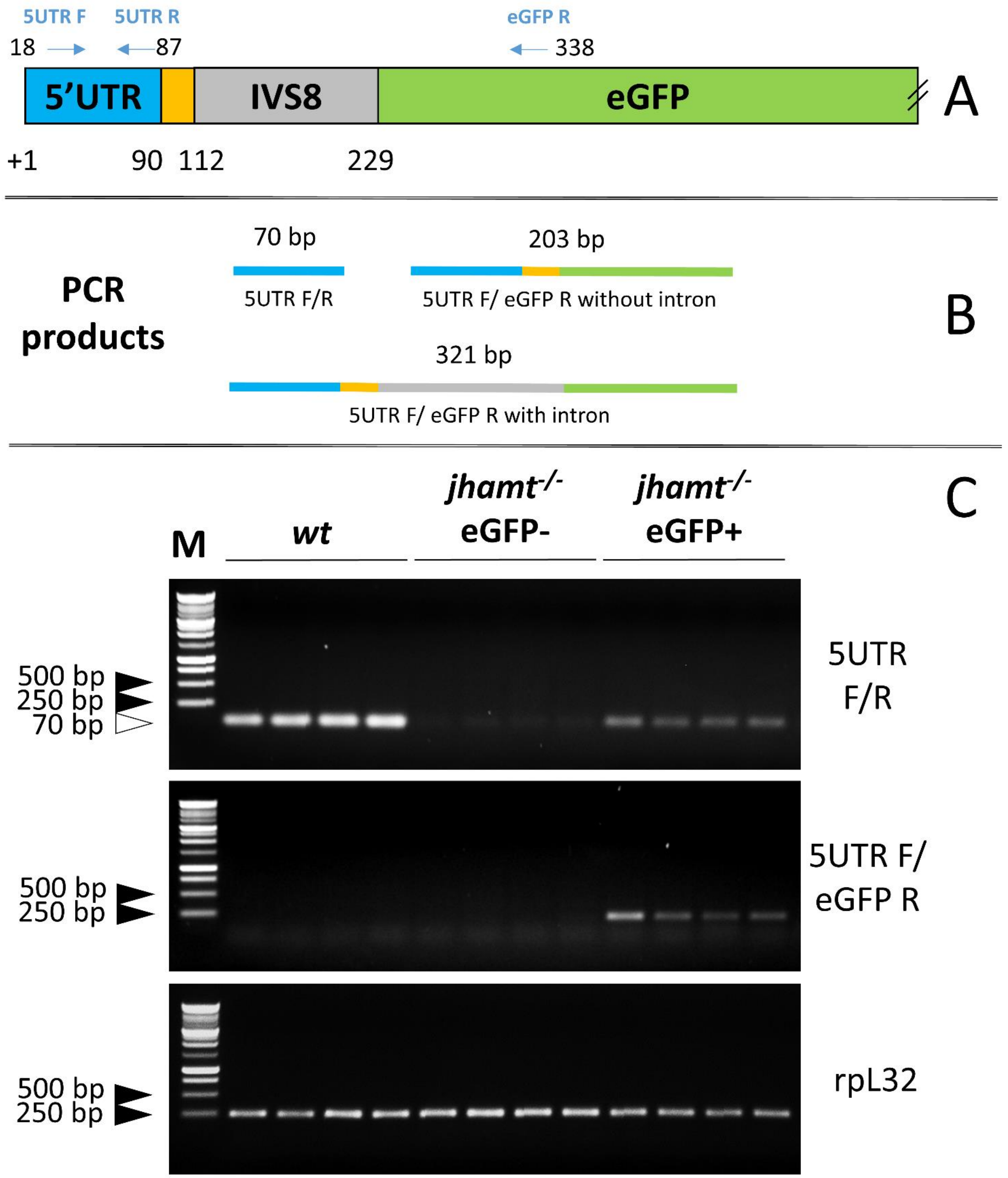

5UTR F
5UTR R
eGFP R
18
87
338
5'UTR
IVS8
eGFP
A
+1
90
112
229
70 bp
203 bp
PCR products
5UTR F/R
5UTR F/ eGFP R without intron
B
321 bp
5UTR F/ eGFP R with intron
C
M
wt
jhamt-/- eGFP-
jhamt-/- eGFP+
500 bp
250 bp
70 bp
5UTR F/R
500 bp
250 bp
5UTR F/ eGFP R
500 bp
250 bp
rpL32

Table S1. **Oligonucleotides and primers**. *, Oligonucleotides used for CRISPR/Cas9-mediated mutagenesis with the target sequence shown in lowercase. **, Primers used for In-Fusion cloning with nucleotides added for the cloning purpose are shown in lowercase; restriction endonuclease cleavage sites are underlined.

**Primer name**	**Sequence (5' → 3')**
JHAMTexon1ACRISPRF*	GAAATTAATACGACTCACTATAGgttagctcggtgataaagattGTTTTAGAGCTAGAAATAGC
JHAMTexon1BCRISPRF*	GAAATTAATACGACTCACTATAGGccaagaacgcgtgcccgtttGTTTTAGAGCTAGAAATAGC
EPOXexon4ACRISPRF*	GAAATTAATACGACTCACTATAGgcttcggtgtagggaagataGTTTTAGAGCTAGAAATAGC
EPOXexon4BCRISPRF*	GAAATTAATACGACTCACTATAggagcgattcctcaacgaaaGTTTTAGAGCTAGAAATAGC
sgRNArev	AAAAGCACCGACTCGGTGCCACTTTTTCAAGTTGATAACGGACTAGCCTTATTTTAACTTGCTATTTCTAGCTCTAAAAC
pSL-dsRed_F**	taacaaccggtacctGGTTTTCCGTGCTCTTTGTC
pSL-dsRed-PmeI_R**	tagctatagttctaggtttaaacTACGCGTATCGATAAGCTTTAAG
pSLNdeI_IVS8_F**	caggcggccgccataGGAACGGTGCATTGGA
EGFP_IVS8_R**	CATGGTGGACCGGTAGCTTG
IVS8_EGFP_F**	TACCGGTCCACCATGGTGAGCAAGG
pSLNdeI_EGFP_R**	ggcctaggatgcataTACGCGTATCGATAAGCTTTAAGATACATTGATGAGT
JHAMT_Upstream_F**	gacacttacctgttaATTAGGTTTGTTCATTTCACTTTCACTCTGAT
JHAMT_Upstream_R**	ggattccccgtgttaCAGGAAGAAAGCTCTGCAGAGAATT
dsRed_JHAMT_DS_F**	tatagttctagGTTTCAATGCTCTACACGAAAATGTGATCAATAGATTGAG
dsRed_JHAMT_DS_R**	cgatacgcgtagtttCGGGCACGCGTTCTTG
SV40_Switch_R	CTAGAGTCCGGAGGATCCTTAGGAG
Swith_SV40_F	tcctccggACTCTAGATCATAATCAGCCATACCACATTTGTAG
1F**	aacgttaacaaccggtttaattaaAGCGGAGTACTGTCCTCCG
1R**	aacaagttcgtcgaCGGCCTGAAGTAAAG
2F**	gtcgacgAACTTGTTTATTGCAGCTTATAATGGTTACAAATAAAGCAA
2R**	ctcaccatGGTGGCGACCGGTGG
3F**	cgccaccATGGTGAGCAAGGGCGAG
3R**	aacaagttttaCTTGTACAGCTCGTCCATGCC

5UTR F	TTGTTCTGGTTTGGCGCATC
5UTR R	ACTTTCACTCTGATTCGTTTTGG
eGFP R	GCTGAACTTGTGGCCGTTTA
rpL32 F	TTGTTCCTTTCTTCCCGAAC
rpL32 R	GTTCGATCCGTAACCGATGT

References cited

1. Mayoral, J.G, Nouzova, M., Yoshiyama, M., Shinoda, T., Hernandez-Martinez, S., Dolghih, E., Turjanski, A.G., Roitberg, A.R., Priestap, H., Perez, M., Mackenzie, L., Li, Y. & Noriega, F.G. Molecular and functional characterization of a juvenile hormone acid methyltransferase expressed in the *corpora allata* of mosquitoes. Insect Bioch. Mol. Biol. 39, 31-37 (2009).

2. Nouzova, M., Edwards, M.J., Mayoral, J.G. & Noriega, F.G. A coordinated expression of biosynthetic enzyme controls the flux of juvenile hormone precursors in the *corpora allata* of mosquitoes. Insect Biochem. Mol. Biol. 41, 660–669 (2011).

3. Nouzova, M., Edwards, M.J., Michalkova, V., Ramirez, C.E., Ruiz, M., Areiza, M., DeGennaro, M., Fernandez-Lima, F., Feyereisen, R., Jindra, M. & Noriega, F.G. Epoxidation of juvenile hormone was a key innovation improving insect reproductive fitness. Proc Natl Acad Sci USA. 118 (45), e2109381118 (2021).

Original gel images requested to be included in the Supplementary Materials by the Quality Control Scientific Reports Editorial Support Staff.

These are the agarose gel images that were utilized to generate the figures included in the supplementary information.

Supplementary Figure 1: The fluorescence of eGFP in early pupae is caused by the perdurance of eGFP protein and not by de novo mRNA transcription.

The top panel from the Supplementary Figure 1 (eGFP) is marked with a dashed rectangle in the original image of the gel displayed below. The middle panel from the Supplementary Figure 1 (JHAMT) is marked with a dotted white rectangle in the original image of the gel displayed below. The bottom panel from the Supplementary Figure 1 (rpL32) is marked with a solid white rectangle in the original image of the gel displayed below. Negative controls are also displayed: -RT (no reverse transcriptase) and NTC (PCR reaction with no template added –no template control).

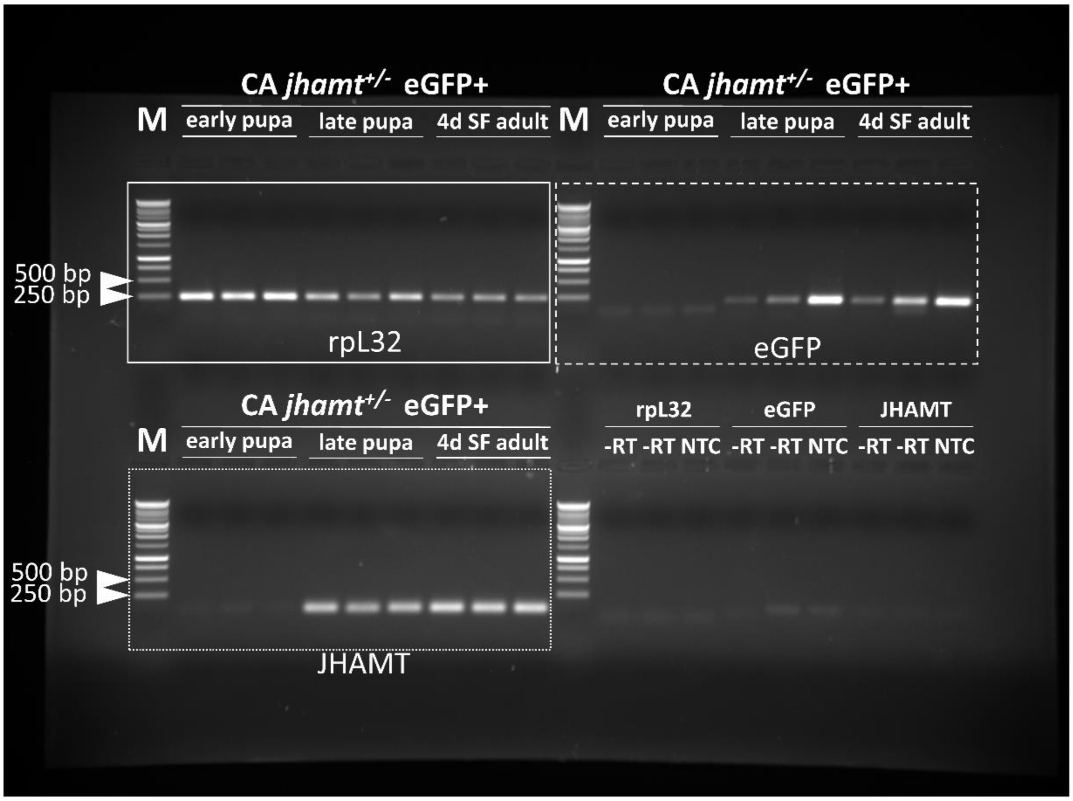

Supplementary Figure 2: Evaluation of alternative splicing due to the presence of the artificial intron IVS8 between the *jhamt* transcription start and the eGFP transgene.

The top panel from the Supplementary Figure 2 (5UTR F/R) is marked with a dashed rectangle in the original image of the gel displayed below. The middle panel from the Supplementary Figure 2 (5UTR F/eGFP R) is marked with a dotted white rectangle in the original image of the gel displayed below. The bottom panel from the Supplementary Figure 2 (rpL32) is marked with a solid white rectangle in the original image of the gel displayed below.

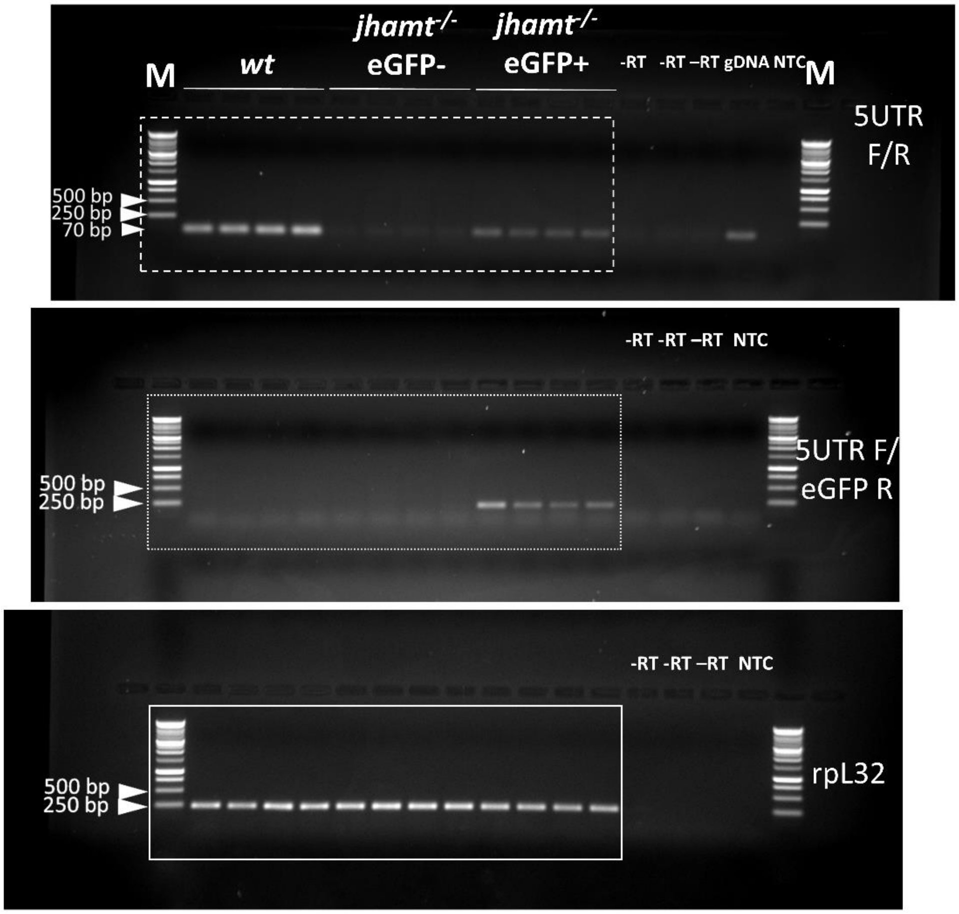

Supplement: Supplementary file 1 — Supplementary Information. [file 41598_2022_25009_MOESM1_ESM.pdf]
